# Massive hematuria due to an autogenous saphenous vein graft and urinary bladder fistula in an extra-anatomic iliofemoral bypass: a case report

**DOI:** 10.1186/s13256-019-2300-8

**Published:** 2019-12-08

**Authors:** Luan Jaha, Vlora Ismaili-Jaha, Bekim Ademi, Fahredin Veselaj, Destan Kryeziu, Bujar Gjikolli, Agreta Gecaj-Gashi, Adhurim Koshi, Art Jaha

**Affiliations:** 1 Department of Vascular Surgery, University Clinical Center of Kosovo, Pristina, Republic of Kosovo; 2 Faculty of Medicine, University “Hasan Prishtina”, Rrethi i Spitalit p.n., 10000 Prishtina, Republic of Kosovo; 3 Department of Urology, University Clinical Center of Kosovo, Pristina, Republic of Kosovo; 4 Department of Radiology, University Clinical Center of Kosovo, Pristina, Republic of Kosovo; 5 Department of Anesthesiology and Intensive Care, University Clinical Center of Kosovo, Pristina, Republic of Kosovo

**Keywords:** Massive hematuria, Autogenous vein graft to urinary bladder fistula, Extra-anatomic bypass

## Abstract

**Introduction:**

Gross hematuria caused by rupture of an artery in the urinary tract is a rare but potentially fatal condition. Iliac artery aneurysms, pelvic surgery with radiation, vascular reconstructive surgery, surgery for stenosis of the ureteropelvic junction, and transplantation are reported to be associated with this condition. In the vascular reconstructive surgery group, the most common etiology is rupture of the degenerated artery or synthetic graft in the ureter.

**Case presentation:**

We present a case of rupture of the small anastomotic pseudoaneurysm at the proximal anastomosis of a right iliofemoral autogenous vein extra-anatomic graft in the urinary bladder. To our knowledge, this is the first report of a rupture of an autogenous vein graft in the urinary bladder. Our patient, a 24-year-old Albanian farmer, was admitted to the emergency department in severe hemorrhagic shock induced by exsanguinating hematuria. He underwent immediate surgery, during which direct sutures to the bladder were placed and the saphenous graft was replaced with a synthetic one. The patient recovered completely, was free of hematuria, and showed no signs of pathological communication between the urinary and arterial tracts on postoperative cystoscopy and computed tomographic angiography during 2 years of follow-up.

**Conclusion:**

The incidence of artery-to-urinary tract fistulas is growing due to the increasing use of urologic and vascular surgery, pelvic oncologic surgery, and radiation therapy. In addition to fistulas involving a degenerated artery and ureter or synthetic grafts and ureter, they can also involve an autogenous vein graft and the urinary bladder. In our patient, the fistula was a result of erosion of the bladder from a pseudoaneurysm at the proximal anastomosis of an autogenous vein iliofemoral bypass in an extra-anatomic position. Open surgery remains the best treatment option, although there is increasing evidence of successful endovascular treatment.

## Introduction

Artery-to-urinary tract fistulas (AUFs) are rare, erosive defects that occur between the segments of the urinary tract and adjacent blood vessels or vascular grafts. From 1908, when first described by Moschcowitz [1], until 10 years ago, less than 150 cases were reported in the literature [2]. Primary fistulas, which account for less than 15% of reported AUFs, are usually associated with arteriovenous malformations and aneurysmal degeneration of the aorta and iliac arteries [3–5], whereas secondary fistulas, which account for all other reported AUFs, are associated with previous vascular surgery, pelvic oncologic surgery, and radiation therapy. All reported fistulas involved exclusively degenerated artery and ureter or synthetic graft and ureter [6–8]. To our knowledge, we report the first instance of the communication of an autogenous vein graft with the bladder.

## Case presentation

Our patient was a 24-year-old Albanian man who was admitted to the Emergency Department of the University Hospital in Kosovo in severe hemorrhagic shock due to a massive hematuria. The patient is a farmer, does not smoke, and does not consume alcohol. He has no significant family and social history of medical relevance. Ten years ago, he had sustained a third-degree burn injury over approximately 70% of his body surface area and had been treated in a specialized center in a neighboring country. During that hospitalization, the patient had multiple venous lines placed in the groin and developed an infection that led to the rupture of the common femoral artery. To treat it, an autogenous vein extra-anatomic iliofemoral bypass was constructed. Since then, he had never been seen by a vascular surgeon.

Prior to his admission to our emergency department, he had two episodes of gross hematuria for which he was treated at the regional hospital. Diagnostic evaluation during earlier hospitalizations did not reveal the cause of bleeding. AUF was not considered on either of the occasions. The treatment was conservative and involved bladder lavage and blood transfusions. He was discharged on antibiotics, uroseptics, and iron supplements.

**Fig. 1:**
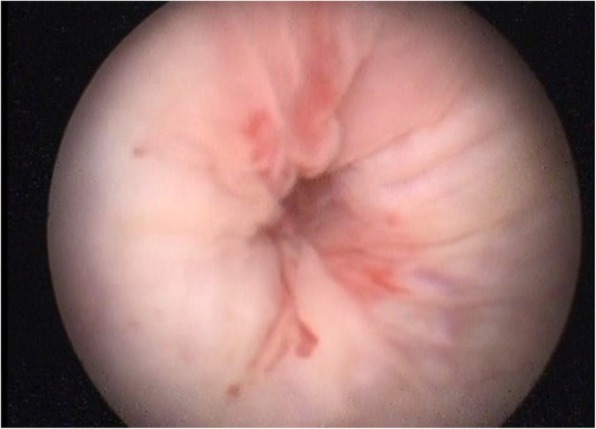
The bleeding site at cystoscopy

**Fig. 2:**
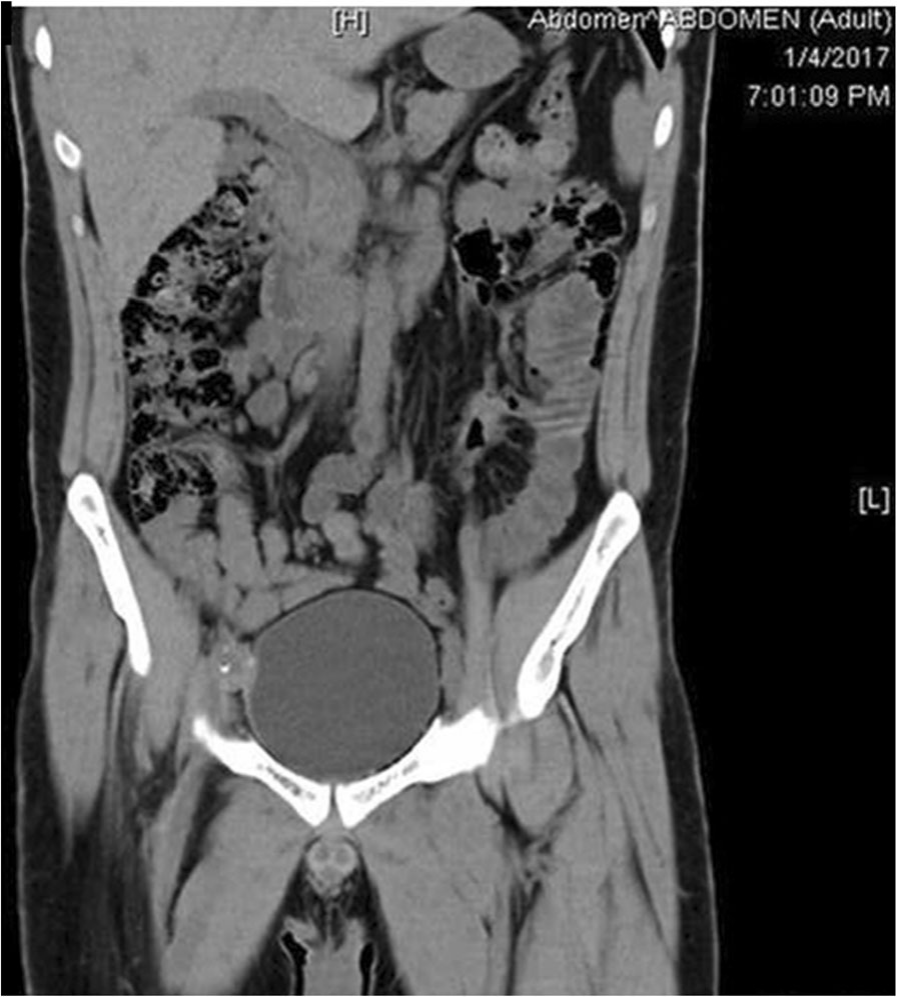
Magnetic resonance imaging showing pseudoaneurysm and the site of rupture at the bladder. Note close proximity of the extra-anatomic graft and urinary bladder

The possibility of communication between the arterial and urinary tracts was suspected on the basis of cystoscopy performed in the outpatient setting 2 days before the current admission (Fig. 1). The finding of the source of the bleeding at the right upper corner of the bladder, very close to the ureteral opening, raised the suspicion of possible AUF. The diagnosis was confirmed the next day, when contrast-enhanced magnetic resonance imaging showed proximity of a small pseudoaneurysm located at the proximal anastomosis of the enlarged extra-anatomic right iliofemoral autogenous vein graft and bladder (Fig. 2). The patient, who was free of bleeding, was referred to a vascular surgeon and admitted to the vascular surgery department. Several units of red blood cells and fresh frozen plasma (FFP) were ordered, along with antibiotics, and the patient was scheduled for elective surgery on the next day. Unfortunately, during the night, he experienced a third episode of exsanguinating bleeding and was transferred to the emergency department.

At the emergency department, he was confused and anxious, and his skin was pale, cold, and clammy. He was sweating and was breathing rapidly. His pulse on the peripheral arteries was weak, rapid, and thready. His fingernails and lips were blue, and his capillary refill time was 5 seconds. His blood pressure was 70/40 mmHg, heart rate 130 beats/minute, and peripheral capillary oxygen saturation 92%. His hematocrit was 19%, and his hemoglobin was 7 g/dl. His platelet count was normal, and his white blood cell count was slightly elevated (11.7 × 10^9^/L). His glucose, cholesterol, urea, and creatinine concentrations were within normal range. His total bilirubin was moderately elevated (30.6 μmol/L), and his transaminase level was normal. He had a significantly high level of C-reactive protein (55.8 mg/L). His urine was full of blood cells. No serology or microbiology was performed. Hemodynamic resuscitation was initiated immediately. Two large-bore (16-gauge) intravenous catheters were inserted. Crystalloids and colloids were rapidly administered, and red blood cells and FFP were ordered.

Induction agents etomidate (0.3 mg/kg), fentanyl (3 μg/kg), and rocuronium (1.2 mg/kg) were administered. The patient was intubated and escorted to the operation room. Anesthesia was maintained with sevoflurane (0.7–1.3 minimum alveolar concentration), atracurium, and fentanyl. To achieve hemodynamic stability, vasopressors (dopamine 5–7 μg/minute) were used until several units of red blood cells and FFP were brought from the transfusion desk. To minimize the possibility of rebleeding, permissive hypotensive resuscitation was maintained.

**Fig. 3:**
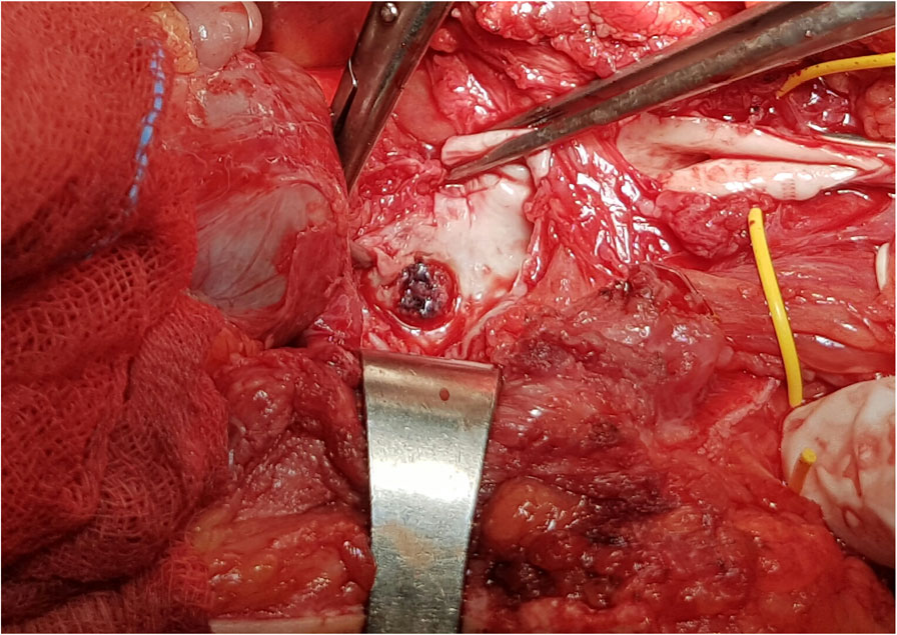
The site of rupture of the anastomotic pseudoaneurysm at the bladder seen through the excised venous graft

**Fig. 4:**
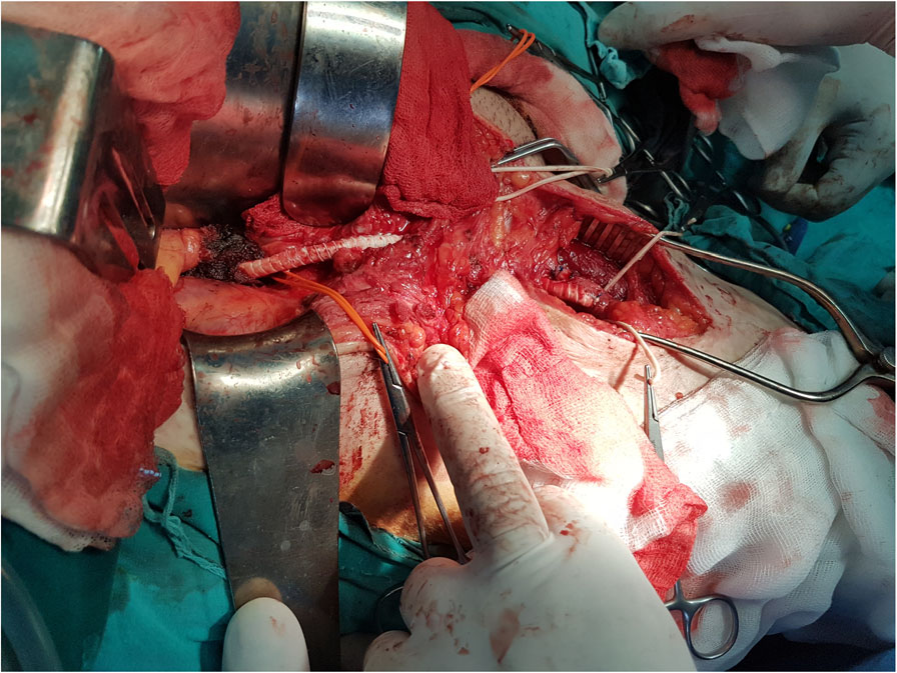
Iliofemoral synthetic bypass. Two anastomoses are shown

The abdomen was opened employing a right extraperitoneal approach. After obtaining vascular control, the rupture site was reached through the native aneurysmal part of the venous graft and was closed with simple sutures (Fig. 3). Because of the severe scars on the skin and varicosity of the saphenous vein, we decided to perform a new bypass using a synthetic graft. The proximal anastomosis of the synthetic graft was placed on the iliac artery 5 cm above the site of the rupture, and the distal part of the graft was anastomosed in an end-to-side fashion with the existing autogenous vein graft, several centimeters before the site of the original distal anastomosis. The graft above the distal anastomosis was ligated (Fig. 4).

**Fig. 5:**
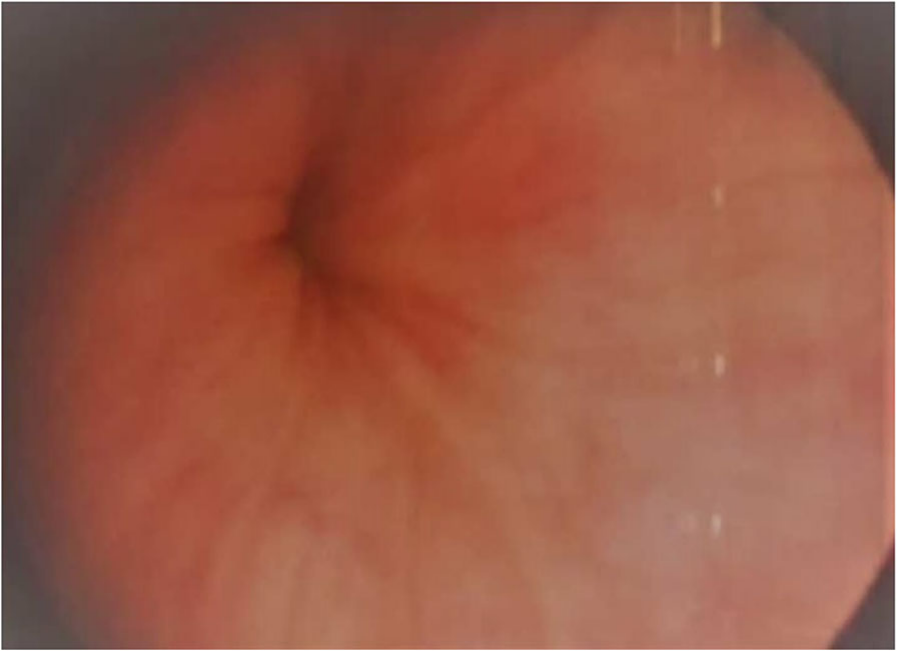
The recovered bleeding site at cystoscopy 3 weeks after the surgery

**Fig. 6:**
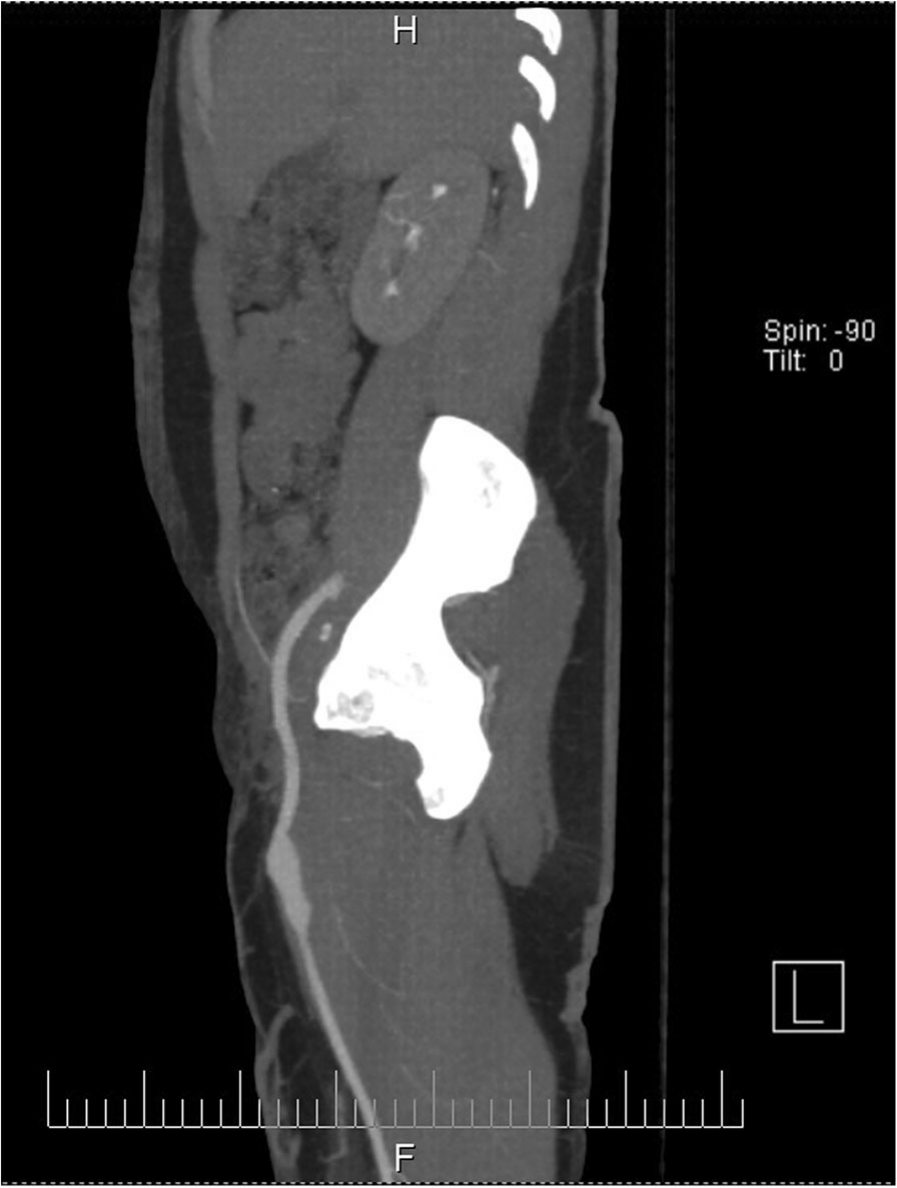
Bypass graft 2 years after the surgery

At the end of the operation, the patient was transferred to the intensive care unit. On the next day, he was extubated and transferred to the ward. The patient recovered completely, and postoperative cystoscopy showed no signs of pathological communication (Fig. 5). He was free of hematuria episodes for the whole postoperative period. Computed angiography performed 2 years after the surgery showed correct position of the graft with no complications (Fig. 6).

## Discussion

Adding to previous reports of AUFs involving a degenerated artery or a synthetic graft with ureter [6–8], we report the first instance of a communication of an autogenous vein graft with bladder. We believe that the reason for this was a small pseudoaneurysm at the site of proximal anastomosis of the autologous vein in an extra-anatomic iliofemoral bypass that eroded the bladder.

There are several theories to explain the development of the pseudoaneurysm [9]. Infection [10–16], degeneration of the artery [10, 12, 17–27], aseptic necrosis at the anastomotic line [28, 29], extensive endarterectomy, [10, 12, 14, 30, 31], large anastomosis or patch [10, 14, 32], the way that the anastomosis was performed [14, 33, 34], the length of the graft [35], adhesion with surrounding structures [34], mismatch between vessels [36–38], and combinations of these situations have been widely discussed. It is not clear which of the aforementioned was responsible for the development of the pseudoaneurysm in our patient, although it is likely that the significantly larger autogenous vein graft may have caused mechanical stress on the proximal anastomosis and contributed to the formation of the pseudoaneurysm. Other contributing factors that should be considered are the extra-anatomic position of the graft and the age of the patient at the time of surgery (14 years old).

Regardless of origin, a typical presentation of AUFs includes different degrees of hematuria ranging from microscopic to exsanguinating hemorrhage. AUFs are found more frequently in women and tend to occur between 2 and 25 years after the initial surgical intervention. Mortality rates of 0–23% have been reported. However, without an accurate preoperative diagnosis, a worse prognosis has been reported (64% mortality rate) [39, 40].

The most common diagnostic tools are ultrasound, computed tomography, and magnetic resonance angiography, and cystoscopy [39, 40]. Surgical management of arterial–ureteral fistulas has several goals, namely hemorrhage control, restoration of vascular and urinary continuity, and resection of potentially infected tissue or prosthetic material [38, 40, 41]. Multiple treatment options regarding the arterial defect have been reported, with a recent trend toward an endovascular approach [42]. Open surgery alternatives are local reconstruction with arteriorrhaphy, patch closure, interposition graft, or bypass. There are reports of simple closure of the rupture site and excision of the graft without reconstruction that have been well tolerated [42–47]. Ureter repair is performed by reimplanting the ureter away from the vessels or by placing a nephrostomy tube. If the kidney is atrophic, nephrectomy and ureterectomy are performed [48].

The first description of endovascular treatment of AUFs was reported by Arap *et al.* [49] in 1965. Since then, multiple options of endovascular treatment of AUFs have been developed, including stent graft exclusion of the fistula and coil embolization of the affected artery with or without placement of a bypass graft for limb perfusion [42, 50–53].

## Conclusion

The incidence of AUFs is growing due to an increase in urologic and vascular surgery, pelvic oncologic surgery, and radiation therapy. In addition to the fistulas involving degenerated artery and ureter or synthetic grafts and ureter, they can also involve autogenous vein graft and urinary bladder. In our patient, the fistula was a result of erosion of the bladder from a pseudoaneurysm at the proximal anastomosis of an autogenous vein iliofemoral bypass in an extra-anatomic position.

Open surgery aimed at hemorrhage control, restoration of vascular and urinary continuity, and resection of potentially infected tissue or prosthetic material remains the best therapeutic option for AUFs, even though there is increasing evidence of successful endovascular treatment.

Early recognition is crucial to success. This is why AUFs should be suspected in all patients with hematuria and a history of the aforementioned surgical and radiological procedures.

## Data Availability

The data are available under consideration of the corresponding author on reasonable request.
